# Hidden Cavities and the Illusion of Margin Sufficiency in Deep Wedge Resection

**DOI:** 10.1093/icvts/ivag116

**Published:** 2026-04-17

**Authors:** Kishankumar Mahida, Snehal Rajendra Jagtap

**Affiliations:** Dr. D. Y. Patil Medical College Hospital and Research Centre, Dr. D. Y. Patil Vidyapeeth (Deemed-to-be-University), Pune, Maharashtra 411018, India; Dr. D. Y. Patil Dental College and Hospital, Dr. D. Y. Patil Vidyapeeth (Deemed-to-be-University), Pune, Maharashtra 411018, India

Dear Editor,

We read with great interest the study by Tokunaga et al., which explores a previously underrecognized mechanism of surgical margin overestimation following deep wedge resection in lung cancer.^1^ The integration of computed tomography imaging of inflated resected specimens with corroborative *ex vivo* porcine compression models provides a compelling cross-validation of the mechanical phenomenon under investigation. The authors’ use of both radiologic and histologic correlates in clinical and experimental settings marks a methodologically robust approach to a highly relevant surgical concern. However, certain interpretive and procedural issues merit further scrutiny in light of the study’s translational ambitions.

A central concern lies in the conceptual framing of margin assessment based solely on resection depth as a predictive surrogate. While the study identifies a threshold depth of 26.3 mm beyond which “empty space” formation becomes likely, this cut-off may conflate depth with a more complex interplay of compressive force distribution, tissue elasticity, and staple configuration. By operationalizing depth as a binary predictor, the risk arises of oversimplifying a 3D mechanical phenomenon, especially given that compression tolerances likely vary across lung zones and parenchymal pathologies.^2^ Clinically, this matters because anatomical location, tissue heterogeneity, and underlying lung disease (such as localized fibrosis or subclinical emphysema) may modulate rupture thresholds independent of depth.

The second interpretive issue concerns the reliance on the number of stapler cartridges as a surrogate for resection complexity. The association between cartridge count and cavity formation is statistically significant, yet this metric does not account for variability in staple height selection, angulation of firing, or the distribution of tissue mass within the jaws. Without standardization of stapling mechanics, cartridge count risks serving as a confounding proxy rather than a reproducible predictor.^3^ Surgeons evaluating margin adequacy intraoperatively may therefore over- or underestimate risk based on a parameter that lacks biomechanical specificity. A more meaningful measure could involve real-time assessment of compression resistance or tissue recoil, which may offer greater fidelity in predicting parenchymal rupture.

Finally, although the *ex vivo* porcine model offers valuable mechanistic insight, its application to surgical decision-making in human segmentectomy requires caution. Lung parenchymal compliance and alveolar integrity in live human tissue—especially in older patients with chronic inflammation or prior interventions—may deviate substantially from porcine lungs immediately post-mortem.^4^ Moreover, the clinical translation of stapler-induced alveolar rupture into oncologic recurrence risk remains speculative without longitudinal follow-up or margin-specific outcome correlation. For surgeons making intraoperative trade-offs between wedge resection and segmentectomy, these gaps may complicate the application of the findings to real-world case selection.

We commend the authors for elucidating a novel and biomechanically plausible mechanism of surgical margin misestimation with potential implications for thoracic oncologic safety. Their findings underscore the need for precision in resection planning, especially when operating in deep parenchymal zones or using multi-cartridge configurations. Future investigations integrating patient-specific tissue characteristics, intraoperative mechanical parameters, and long-term oncologic outcomes will be critical in determining whether compression-induced empty space formation warrants a shift in procedural thresholds or surgical algorithms.

## Data Availability

Not applicable, as no data were generated or analyzed in this study.
